# Diagnostic yield of array CGH in patients with autism spectrum disorder in Hong Kong

**DOI:** 10.1186/s40169-016-0098-1

**Published:** 2016-05-16

**Authors:** Wai-Kwan Siu, Ching-Wan Lam, Chloe Miu Mak, Elizabeth Tak-Kwong Lau, Mary Hoi-Yin Tang, Wing-Fai Tang, Rachel Sui-Man Poon-Mak, Chi-Chiu Lee, Se-Fong Hung, Patrick Wing-Leung Leung, Karen Ling Kwong, Eric Kin-Cheong Yau, Grace Sui-Fun Ng, Nai-Chung Fong, Kwok-Yin Chan

**Affiliations:** Department of Pathology, The University of Hong Kong, 102 Pokfulam Road, Hong Kong, China; Kowloon West Cluster Laboratory Genetics Service, Department of Pathology, Princess Margaret Hospital, Hong Kong, China; Department of Obstetrics and Gynaecology, The University of Hong Kong, Queen Mary Hospital, Hong Kong, China; Department of Clinical Psychology, Kwai Chung Hospital, Hong Kong, China; Department of Psychiatry, Kwai Chung Hospital, Hong Kong, China; Department of Psychology, The Chinese University of Hong Kong, Hong Kong, China; Department of Paediatrics and Adolescent Medicine, Tuen Mun Hospital, Hong Kong, China; Department of Paediatrics and Adolescent Medicine, Princess Margaret Hospital, Hong Kong, China

**Keywords:** Autism spectrum disorder, Chinese, ARRAY CGH

## Abstract

**Background:**

Chromosomal microarray offers superior sensitivity for identification of submicroscopic copy number variants (CNV) and it is advocated to be the first tier genetic testing for patients with autism spectrum disorder (ASD). In this regard, diagnostic yield of array comparative genomic hybridization (CGH) for ASD patients is determined in a cohort of Chinese patients in Hong Kong.

**Methods:**

A combined adult and paediatric cohort of 68 Chinese ASD patients (41 patients in adult group and 27 patients in paediatric group). The genomic DNA extracted from blood samples were analysed by array CGH using NimbleGen CGX-135K oligonucleotide array.

**Results:**

We identified 15 CNV and eight of them were clinically significant. The overall diagnostic yield was 11.8 %. Five clinically significant CNV were detected in the adult group and three were in the paediatric group, providing diagnostic yields of 12.2 and 11.1 % respectively. The most frequently detected CNV was 16p13.11 duplications which were present in 4 patients (5.9 % of the cohort).

**Conclusions:**

In this study, a satisfactory diagnostic yield of array CGH was demonstrated in a Chinese ASD patient cohort which supported the clinical usefulness of array CGH as the first line testing of ASD in Hong Kong.

**Electronic supplementary material:**

The online version of this article (doi:10.1186/s40169-016-0098-1) contains supplementary material, which is available to authorized users.

## Background

Autism spectrum disorder (ASD) is a collective term describing a range of neurodevelopmental disorders with core features of deficits in communications and social interactions, accompanied by stereotyped behaviours and restricted interest. The global prevalence was reported to be 1 in 161 children, affecting more males than females [1]. Being regarded as a crucial factor for the aetiology of ASD, genetic alterations identified in affected patients are remarkably heterogeneous across the whole genome [2].

Evidently, a number of chromosomal abnormalities have been recognised to be associated with ASD phenotype [3]. Nevertheless, the diagnostic yield of conventional G-banded karyotype has been reported to be only 3 % [4]. Notably, chromosomal microarray (CMA) is regarded as a robust and comprehensive tool for genome-wide detection of submicroscopic deletions and duplications, which are named as copy number variants (CNV). The advantage of high resolution using CMA translates into major improvement in the detection rate. Indeed, the implications of rare CNV on the pathogenesis of ASD have been increasingly acknowledged [5]. CMA is now regarded as the first tier genetic testing for ASD patients [6]. The diagnostic importance of CMA for ASD has been demonstrated in diverse clinical settings [7–9]. Overall, the frequency of finding clinically significant CNV in ASD patients has been shown to be approximately 7–9 % [10, 11]. Moreover, Tammimies et al. has demonstrated that the diagnostic yield was significantly higher in those with more complex morphological phenotype [12]. Nevertheless, in majority of the CNV studies in ASD, the subjects are predominately from Caucasian ancestry. Seemingly, genomic data in other population is crucial, especially when CMA are increasingly adopted in clinical laboratories.

In this study, we determined the clinical usefulness of CMA in evaluation of ASD patients in our population. Array comparative genomic hybridization (CGH) is the platform that used for identification of CNV in a Chinese ASD patient cohort from Hong Kong. We present here the diagnostic yield of this investigative tool in a combined adult and paediatric cohort.

## Methods

### Patients

We evaluated a combined adult and paediatric cohort of 68 patients (60 males and 8 females). All the patients are unrelated. The adult patients were recruited from a cohort of a local study on the adult outcome of children with autism with normal intelligence [13]. Forty-one patients were in the adult group (39 males and 2 females) and the age was 22–33 years (median 27 years). This group consisted of ASD patients who were diagnosed in childhood by psychiatrists, paediatricians or clinical psychologists before year 1990, using the Diagnostic and Statistical Manual of Mental Disorders, Third Revised Edition, and confirmed with the development, dimensional and diagnostic interview [14] during adulthood in the aforementioned study. The adult cohort was also assessed with Wechsler Adult Intelligence Scale-Third Edition (WAIS-III) Chinese version [15] and confirmed to have normal intelligence with full IQ score of 75 or above. They had follow-up in clinic under Kwai Chung Hospital. In the paediatric group, 27 patients were recruited (21 males and 6 females), aged 2–15 (median 5 years). The paediatric patients were assessed in the Department of Paediatrics and Adolescent Medicine of Princess Margaret Hospital or Tuen Mun Hospital. The paediatric patients were assessed using autism diagnostic interview-revised (ADI-R) [16] to confirm the diagnosis of ASD. Thirteen patients in the paediatric group (52.0 %) also had developmental delay. The study was approved by the Clinical Research Ethic Committee of Kowloon West Cluster & New Territories West Cluster of Hospital Authority (Reference number: KWC/FR/10-007 and NTWC/CREC/1004/11). Informed consent was obtained from all parents or patients.

### Array CGH and data interpretation

Peripheral blood samples were collected in EDTA tubes for genomic DNA extraction using QIAamp Blood Kit (Qiagen, Hilden, Germany). The quantity of DNA in the samples was measured by Nanodrop spectrophotometer and all samples had an A_260_/A_280_ ratio more than or equal to 1.8. Agarose gel electrophoresis was used for the assessment of DNA quality to preclude any degradation or RNA contamination. NimbleGen CGX-135K oligonucleotide arrays [Genome Build: hg18] were used in this study and the method was previously described [17]. This platform had been used in multiple clinical microarray studies [18–21]. The data was analysed using DEVA (Roche NimbleGen, Wisconsin, USA) and Genoglyphix (Signature Genomics, Spokane, USA). The quality of array CGH experiments has been assessed through the parameters in the quality metric report. The reports include “signal range” and “ratio range” which represent the uniformity of log-2 ratio over the array. The lower the value, the better the quality of the data. The array CGH data of all samples had “signal range” and “ratio range” below the cutoffs suggested by the manufacture which were <1.0 and <1.5 respectively.

The clinical significance of the CNV detected was determined using the information available in the open access databases including Database of Chromosomal Imbalance and Phenotype in Human using Ensembl Resources (DECIPHER), Database of Genomic Variant (DGV), International Standards for Cytogenomic Arrays Consortium Database (ISCA), and Simons Foundation Autism Research Initiative Gene (SFARI Gene). Categorization of CNV is based on available information on the clinical significance of genes in the region of deletions or duplications via the search in The University of California Santa Cruz (UCSC) Genome Browser, Pubmed and Online Mendelian Inheritance in Man (OMIM). CNV are classified into pathogenic, uncertain clinical significance and benign based on American College of Medical Genetics guideline [22] and the pathogenic and likely pathogenic CNV are deemed to be clinically significant. Detection rate was defined as the number of patients with CNV divided by the total number of patients tested and diagnostic yield was determined as the percentage of patients with clinically significant CNV among patients tested.

## Results

Table 1 shows the summary of the patient characteristics and CNV findings. We identified 15 CNV in the cohort 68 ASD patients, giving CNV detection rate of 22.1 %. Among patients with CNV, there were 13 males and two females. The CNV detection rates in male and female patients were 21.7 and 25 % respectively. In the adult group, CNV were detected in 8 male patients. The overall CNV detection rate in the adult group was 19.5 % for all adults and 20.5 % for male adults. Seven patients with CNV were from the paediatric group with five males and two female. The overall CNV detection rate in the paediatric group was 25.9 % (23.8 % for male and 33.3 % for female).

**Table 1:** Summary of patient characteristics and CNV findings

	Overall	Adult group	Paediatric group
Number of patients	68	41	27
Male	60	39	21
Female	8	2	6
Age range (median) [years]	2–33 (25)	22–33 (27)	2–15 (5)
Intelligence quotient (median)	N/A	75–129 (96)	N/A
Total number of CNV	15	8	7
(Detection rate %)	(22.1)	(19.5)	(25.9)
Detected in male	13	8	4
(Detection rate %)	(21.7)	(20.5)	(19.0)
Detected in female	2	0	3
(Detection rate %)	(25)	(0)	(50)
Clinical significant CNV	8	5	3
(Diagnostic yield %)	(11.8)	(12.2)	(11.1)
Detected in male	6	5	1
(Diagnostic yield %)	(10)	(12.8)	(4.8)
Detected in female	2	0	2
(Diagnostic yield %)	(25)	(0)	(33.3)

*N*/*A* not available

Among the detected CNV, eight of them were classified as clinically significant, which gives an overall diagnostic yield of 11.8 %. Five was from the adult group and three was the paediatric group and the diagnostic yield was 12.2 and 11.1 % respectively. All patients with clinically significant CNV in the adult group were male. In the paediatric group, the clinically significant CNV were found in one male and two female patients. Variants of uncertain clinical significance (VOUS) were detected in seven patients, which contributed to 10.3 % of the entire cohort. The detected CNV were compared to the published CNV map of the human genome [23]. The array CGH data was shown in Additional file 1: Figure S1–S12.

The eight clinically significant CNV contributed to 53.3 % of all CNV (8 out of 15 CNV) detected. Four of them were deletions and another four are duplications. The largest clinically significant CNV identified sized 14.53 Mb while the smallest was 0.12 Mb. Seven of them were below 5 Mb (87.5 %) which was the size range not routinely detectable by karyotype. The size of five CNV was between 1 and 5 Mb (62.5 %) and two were <1 Mb (25.0 %).

The clinically significant CNV were listed in Table 2. Among the clinically significant CNV, 1.16 Mb microduplications within chromosome band 16p13.11 were most frequently observed and these were detected in three adult patients and one paediatric patient. In the adult group, one patient had 1.97 Mb microdeletion at 15q23–q24.1 encompassing 19 genes. Another had 0.26 Mb microdeletion within chromosome band 15q11.2 overlapping Prader-Willi/Angelman region and involving *NIPA1* gene. In the paediatric group, one patient had a large terminal deletion of chromosome 18 from band q22.1 to q23 which is 14.53 Mb in size. Another paediatric patient had 14q22.1 microdeletion involving the whole *NIN* gene.

**Table 2:** Clinically significant CNV

Patient number	Gender/group	Array CGH result [hg18]	Chromosome region	Aberration type	Size (Mb)	Clinical significance	IQ	Additional clinical features
1	Male/adult	arr 16p13.11(15,033,259–16,195,404) × 3	16p13.11	Duplication	1.16	Susceptibility to ASD	82	Nil
2	Male/adult	arr 15q11.2(20,372,901–20,636,841) × 1	15q11.2	Deletion	0.26	Deletion in Prader-Willi/Angelman region with *NIPA1* gene involved	129	Nil
3	Male/adult	arr 15q23q24.1(69,471,038–71,439,732) × 1	15q23–q24.1	Deletion	1.97	Deletion of 19 genes. Known association with ASD and intellectual disability	85	Subtle facial dysmorphism
4	Male/adult	arr 16p13.11(15,033,259–16,195,404) × 3	16p13.11	Duplication	1.16	Susceptibility to ASD	90	Nil
5	Male/adult	arr 16p13.11 (15,033,259–16,195,404) × 3	16p13.11	Duplication	1.16	Susceptibility to ASD	96	Left frontal haemangioma, epilepsy
6	Male/paediatric	arr 14q22.1(50,241,594-50,360,747) × 1	14q22.1	Deletion	0.12	Deletion of the *NIN* gene	N/A	Nil
7	Female/paediatric	arr 18q22.1q23(61,576,686–76,114,624) × 1	18q22.1–q23	Deletion	14.53	Large terminal deletion	N/A	Developmental delay, hypotonia, hearing loss, delayed myelination of brain, umbilical hernia and ear canal stenosis
8	Female/paediatric	arr 16p13.11(15,033,259–16,195,404) × 3	16p13.11	Duplication	1.16	Susceptibility to ASD	N/A	Developmental delay

*N*/*A* not available

For the seven VOUS, six were duplications and one was deletion. All of them were less than 5 Mb. The size of VOUS ranged from 0.08 to 0.97 Mb. The VOUS are listed in Table 3.

**Table 3:** List of variants of uncertain significance

Patient number	Gender/group	Array CGH result [hg18]	Chromosome region	Aberration type	Size (Mb)	IQ	Additional clinical features
9	Male/adult	arr 3q13.3 (111,747,166–112,297,084) × 3	3q13.3	Duplication	0.55	96	Nil
10	Male/adult	arr 1q44 (244,474,644–245,087,421) × 3	1q44	Duplication	0.61	85	Nil
11	Female/adult	arr 11q24.1 (122,330,312–122,406,276) × 3	11q24.1	Duplication	0.08	103	Nil
12	Female/paediatric	arr 10p12.33p12.32 (19,502,326–20,471,711) × 3	10p12.33–p12.32	Duplication	0.97	N/A	Scoliosis
13	Male/paediatric	arr 17q21.33 (45,861,307–45,986,282) × 3	17q21.33	Duplication	0.12	N/A	Developmental delay
14	Male/paediatric	arr 6q14.1 (82,900,869–83,543,710) × 3	6q14.1	Duplication	0.64	N/A	Developmental delay/regression, asthma, severe eczema
15	Male/paediatric	arr 5q33.1 (148,226,533–148,809,596) × 1	5q33.1	Deletion	0.58	N/A	Nil

*N*/*A* not available

## Discussion

We identified 11.8 % patients with clinically significant CNV in our ASD cohort by array CGH. The diagnostic yield in this study was in keeping with other studies [11, 24]. If conventional G-banded karyotype was used as the first line test, only one patient in our cohort (1.5 %) would have chromosomal abnormality detected microscopically. With high resolution, array CGH is capable of identifying the underlying chromosomal cause of ASD in a much greater number of patients. Evidently, our results demonstrated a satisfactory diagnostic yield of array CGH for genetic diagnosis in ASD patients, confirming its clinical usefulness as first tier testing. The diagnostic yield of the in the adult and paediatric group was comparable.

In addition, array CGH also allowed better delineation of the breakpoints of the CNV. The improved accuracy facilitated genotype-phenotype correlation and identification of candidate genes [6]. In the patient with 15q11.2 deletion at Prader-Willi/Angelman region, the deletion overlapped with reported microdeletion at 15q11.2 between breakpoint (BP) 1 to 2 which was a susceptibility region for autism and language delay [25, 26]. The phenotype of this BP1–BP2 microdeletion is different from those with deletions with proximal breakpoint at BP1 or BP2 and distal breakpoint at BP3 which result in classical Prader-Willi/Angelman syndrome. In the patient with deletion at 15q23–q24.1, the deletion overlapped with previously reported 15q24 deletion in ASD patients [27, 28]. The improved breakpoint delineation drove the identification of novel disease gene *NEO1* which conferred aetiological importance in ASD [29].

The largest CNV detected in this cohort was a deletion within chromosome band 18q21.1q23. The deletion, arr 18q22.1q23 (61,576,686–76,114,624) × 1, encompassed a minimum size of 14.53 Mb and involved 38 genes from *CDH7* to *PARD6G* (Additional file 1: Figure S13). Deletions of 18q were deemed to be a particularly heterogeneous genomic disorder as no recurrent breakpoints were identified [30]. With remarkable genomic heterogeneity, the phenotypes of patients with 18q deletion were highly variable. Thus, it was not feasible to derive this condition based on a collection of clinical characteristics, and genomic analysis would be indispensable for the diagnosis. Clinically, our patient had developmental delay, hypotonia, hearing loss, delayed myelination of the brain, umbilical hernia and ear canal stenosis which were deemed to be core features of distal 18q deletion [31]. In addition, congenital cardiac anomalies were also one of clinical characteristics of 18q deletion that were present in up to 54 % patients [31–33]. For our patient, the echocardiogram was normal. Particularly, constitutional hemizygosity of 18q has been reported to confer increased risk of autism. Forty-three percent of patients with 18q deletion were categorised to be at risk of autism and the likelihood was significantly increased when *TCF4, NETO1* and *FBXO15* were included in the region of hemizygosity [34]. In our patient, *NETO1* and *FBXO15* were included in the deletion. Nevertheless, no shared region of deletion has been identified among the autistic patients with 18q deletion. Hence, the genetic determinants of autism in this group of patients were yet to be elucidated.

The 16p13.11 duplications were the most frequent clinically significant CNV identified in our cohort. Four patients carried the 16p13.11 duplications and all of them shared the same breakpoints at position 15.03 to 16.20 Mb. This represented 5.9 % of our ASD cohort. The main mechanism underpinning the recurrent duplications and deletions at 16p13.11 is the non-allelic homologous recombination occurring between low copy repeats. The recurrent 16p13.11 duplications have established association with autism [35, 36] and a wide range of neuropsychiatric disorders including schizophrenia [37], attention-deficit hyperactivity disorder [38], and intellectual disability [39]. Inheritance from unaffected or mildly affected parents has been reported for the 16p13.11 duplications, indicating incomplete penetrance [35, 36].

Indeed, the effect of 16p13.11 duplications is not without controversy as its frequency in normal population is >1 % and thus being regarded as a common CNV. These duplications have been considered to be benign or uncertain significance in certain studies [40, 41]. Nevertheless, two large surveys on case–control cohorts, with case numbers of 10,397 and 29,085 respectively, have consistently demonstrated 16p13.11 duplications predisposing to ASD and other kinds of neurodevelopmental disorders with statistically significant odd ratios [42, 43]. In the earlier study for one of the aforementioned survey, the odd ratio did not reach the statistical significance with the case number of 15,767 [44]. This illustrates that a remarkably large sample size is required to demonstrate the effect of these alleles with reduced penetrance. It has been recognised that common variations are accountable for majority of the genetic risk for ASD [45]. Seemingly, this susceptibility allele has an indisputable role in the genetic architecture of autism [43]. For interpretation in individual patients, prudent judgment should be exercised for the evaluation of the CNV with reduced penetrance and complete interpretation should be made in the context of phenotypic evaluation and establishing inheritance pattern.

Interestingly, the detection rate of 16p13.11 duplications was relatively high in this study comparing to other CNV studies of ASD. Although CNV data in population-matched controls was not available in the study, the CNV map of the human genome showed that 16p13.11 duplications were not particularly prevalent in Asian comparing to the other ethnic group [23]. Furthermore, the high proportion of duplications detected might be related the clinical characteristics of the adult cohort which they had severe impairment related to autistic symptoms but normal intelligence. It has been reported that duplications correlated to autism severity while deletions had impact on nonverbal IQ [46]. This might explain why of duplications were dominated in the adult cohort of this study. In addition, due to the uncertainty of the effect of 16p13.11 duplications, the possibility of underreporting of this CNV in other autism studies could not be excluded.

One patient had a deletion at chromosome 14q22.1 involving the *NIN* gene. Compound heterozygous mutations of *NIN* gene were reported in microcephalic primordial dwarfism disorder [47]. We sequenced all the coding exons and flanking regions of *NIN* gene but did not reveal any other pathogenic mutations (data not shown). Clinically, the patient had normal growth and no dysmorphic features. *NIN* gene encoded for ninein, a centrosomal protein involved in microtubule anchoring. In the absence of ninein, the progenitors were prematurely depleted at the ventricular zone of the developing mammalian neocortex [48]. Having played a crucial role on microtubule stability, ninein had significant impact on the axonal development and bifurcation [49]. Disruptions of neocortex development and axon guidance were proven to be pivotal in the pathophysiology of ASD [50–53]. Those findings on the function of ninein in brain development indicated the possible link between *NIN* gene and autism. As exemplified by *CNTNAP2 and NRXN1* gene, heterozygous missense variants confer susceptibility to autism [54, 55] while compound heterozygous mutations of *CNTNAP2* and *NRXN1* cause Pitts-Hopkins like syndrome [56]. Moreover, deletion in this region has not been reported in any normal subjects from the DGV database. Therefore, we classified this deletion as clinically significant and *NIN* gene might be considered to be a potential candidate gene for ASD.

This study demonstrated the spectrum of CNV in Chinese ASD patients from Hong Kong and they showed differences in certain aspects comparing with the CNV data from other studies which were mainly from European ancestry [5, 12, 40, 46]. Similar to the other studies, the CNV detected were majority on the hotspots with recurrent breakpoints but the proportion was higher in this study. The CNV in hotspot with recurrent breakpoints generally accounted for 37–53 % of CNV detected in the ASD patients in previous studies [12, 40] and the proportion was 62.5 % (5 out of 8 clinically significant CNV) in this cohort. Moreover, CNV in 16p11.2 was demonstrated to be the most commonly detected CNV in ASD patients but it was still found in largely below 1 % of ASD subjects [12, 27, 40, 46]. In contrast, the most frequently detected CNV in this cohort was 16p13.11 duplications, which were present in 5.9 % of ASD patients. The reason underpinning such a high detection rate has not yet been fully elucidated but this should be validated in a larger Chinese cohort and compared with controls from the local population. The yield of highly penetrant CNV was also relatively low in this study. This could be related to the clinical characteristics of this cohort. From the review of medical record, most of the patients did not have additional clinical features apart from autism. The presence of other physical anomalies has been shown to result in higher diagnostic yield [12]. Furthermore, there was CNV disrupting genes that had not yet described to be linked to ASD. This concurred with other evidence showing numerous genes associated with ASD scattered across the genome and many of ASD risk genes remained to be identified.

In this study, VOUS accounted for 46.7 % of all CNV detected (7 out of 15 CNV). All VOUS were less than 1 Mb in size and majority of them were duplications. Parental results might facilitate the interpretation of the VOUS but the parental samples were not available during the recruitment which represented a limitation of this study. Yet, inheritance from parents would not completely diminish the clinical significance of variants with incomplete penetrance, like the 16p13.11 duplications. In addition, the small size of the duplications would not entirely preclude pathogenic effects. Indeed, evidence for dosage pathogenicity of genes in those regions would be a more important factor to be considered. To exemplify, duplication of a single gene could result in severe phenotype like in *MECP2* duplication syndrome which was associated with autism and mental retardation [57]. Therefore, these VOUS might still deserve further investigations for any possible association with ASD.

It was acknowledged that limitation existed in this study. In terms of number of patients, it was relatively small in this cohort. The retrospective cohort study design also made it prone to selection bias. Moreover, this retrospective cohort of patients with confirmed diagnosis of ASD did not have systematic phenotypic documentation in details. Thus, this restricted the establishment of genotype and phenotype correlation and stratification of the diagnostic yield in patients according to additional clinical features. Another deficiency of this study is the absence of parental samples which could be helpful for the determination of the inheritance to aid the interpretation particularly for VOUS. In addition, control CNV data from normal individuals in the same population were lacking in the present study. The findings from this study should be validated in a larger Chinese ASD cohort from Hong Kong with CNV data from population-matched controls for interpretation. On the analytical aspect, the CNV findings were not checked with a second method. However, all the raw data of each CNV was manually inspected and passed the recommended quality parameters.

## Conclusions

Our study demonstrated a satisfactory diagnostic yield of array CGH in a Chinese ASD patient cohort. Having high resolution for CNV detection, array CGH made a sizeable difference to delineate the genomic alterations in ASD patients. Seemingly, the results supported the clinical usefulness of array CGH as the first tier test of ASD in Hong Kong.

## Additional file

10.1186/s40169-016-0098-1 Figures of array CGH data.

## Abbreviations

CGH: comparative genomic hybridization
ASD: autism spectrum disorder
CMA: chromosomal microarray
CNV: copy number variants
VOUS: variants of uncertain significance

## References

[1] Elsabbagh M, Divan G, Koh YJ, Kim YS, Kauchali S, Marcin C (2012). Global prevalence of autism and other pervasive developmental disorders. Autism Res.

[2] Chang J, Gilman SR, Chiang AH, Sanders SJ, Vitkup D (2015). Genotype to phenotype relationships in autism spectrum disorders. Nat Neurosci.

[3] Vorstman JA, Staal WG, van Daalen E, van Engeland H, Hochstenbach PF, Franke L (2006). Identification of novel autism candidate regions through analysis of reported cytogenetic abnormalities associated with autism. Mol Psychiatry.

[4] Reddy KS (2005). Cytogenetic abnormalities and fragile-X syndrome in autism spectrum disorder. BMC Med Genet.

[5] Pinto D, Pagnamenta AT, Klei L, Anney R, Merico D, Regan R (2010). Functional impact of global rare copy number variation in autism spectrum disorders. Nature.

[6] Miller DT, Adam MP, Aradhya S, Biesecker LG, Brothman AR, Carter NP (2010). Consensus statement: chromosomal microarray is a first-tier clinical diagnostic test for individuals with developmental disabilities or congenital anomalies. Am J Hum Genet.

[7] Roberts JL, Hovanes K, Dasouki M, Manzardo AM, Butler MG (2014). Chromosomal microarray analysis of consecutive individuals with autism spectrum disorders or learning disability presenting for genetic services. Gene.

[8] Nicholl J, Waters W, Mulley JC, Suwalski S, Brown S, Hull Y (2014). Cognitive deficit and autism spectrum disorders: prospective diagnosis by array CGH. Pathology.

[9] Battaglia A, Doccini V, Bernardini L, Novelli A, Loddo S, Capalbo A (2013). Confirmation of chromosomal microarray as a first-tier clinical diagnostic test for individuals with developmental delay, intellectual disability, autism spectrum disorders and dysmorphic features. Eur J Paediatr Neurol.

[10] McGrew SG, Peters BR, Crittendon JA, Veenstra-Vanderweele J (2012). Diagnostic yield of chromosomal microarray analysis in an autism primary care practice: which guidelines to implement?. J Autism Dev Disord.

[11] Shen Y, Dies KA, Holm IA, Bridgemohan C, Sobeih MM, Caronna EB (2010). Clinical genetic testing for patients with autism spectrum disorders. Pediatrics.

[12] Tammimies K, Marshall CR, Walker S, Kaur G, Thiruvahindrapuram B, Lionel AC (2015). Molecular diagnostic yield of chromosomal microarray analysis and whole-exome sequencing in children with autism spectrum disorder. JAMA.

[13] Poon Mak SM (2009) Adult Outcome of Children with autism with normal intelligence Hong Kong: The Chinese University of Hong Kong

[14] Lai KY, Leung PW, Mo FY, Lee MM, Shea CK, Chan GF (2015). Validation of the developmental, dimensional and diagnostic interview (3Di) among Chinese children in a child psychiatry clinic in Hong Kong. J Autism Dev Disord.

[15] Wechsler D (2002). Wechsler adult intelligence scale-3rd edition (Chinese Version).

[16] Rutter M, Le Couteur A, Lord C (2003). Autism diagnostic interview-revised (ADI–R) manual.

[17] Kan AS, Lau ET, Tang WF, Chan SS, Ding SC, Chan KY (2014). Whole-genome array CGH evaluation for replacing prenatal karyotyping in Hong Kong. PLoS ONE.

[18] Rim JH, Kim SW, Han SH, Yoo J (2015). Clinical and molecular delineation of a novel de novo 4q28.3-31.21 interstitial deletion in a patient with developmental delay. Yonsei Med J.

[19] Kim J, Won HH, Kim Y, Choi JR, Yu N, Lee KA (2015). Breakpoint mapping by whole genome sequencing identifies PTH2R gene disruption in a patient with midline craniosynostosis and a de novo balanced chromosomal rearrangement. J Med Genet.

[20] Genesio R, Fontana P, Mormile A, Casertano A, Falco M, Conti A (2015). Constitutional chromothripsis involving the critical region of 9q21.13 microdeletion syndrome. Mol Cytogenet.

[21] Beke A, Piko H, Haltrich I, Csomor J, Matolcsy A, Fekete G (2013). Molecular cytogenetic analysis of Xq critical regions in premature ovarian failure. Mol Cytogenet.

[22] Kearney HM, Thorland EC, Brown KK, Quintero-Rivera F, South ST (2011). Working group of the american college of medical genetics laboratory quality assurance C. American College of Medical Genetics standards and guidelines for interpretation and reporting of postnatal constitutional copy number variants. Genetics in medicine: official journal of the American College of Medical. Genetics.

[23] Zarrei M, MacDonald JR, Merico D, Scherer SW (2015). A copy number variation map of the human genome. Nat Rev Genet.

[24] Sebat J, Lakshmi B, Malhotra D, Troge J, Lese-Martin C, Walsh T (2007). Strong association of de novo copy number mutations with autism. Science.

[25] Burnside RD, Pasion R, Mikhail FM, Carroll AJ, Robin NH, Youngs EL (2011). Microdeletion/microduplication of proximal 15q11.2 between BP1 and BP2: a susceptibility region for neurological dysfunction including developmental and language delay. Hum Genet.

[26] Doornbos M, Sikkema-Raddatz B, Ruijvenkamp CA, Dijkhuizen T, Bijlsma EK, Gijsbers AC (2009). Nine patients with a microdeletion 15q11.2 between breakpoints 1 and 2 of the Prader-Willi critical region, possibly associated with behavioural disturbances. Eur J Med Genet.

[27] Marshall CR, Noor A, Vincent JB, Lionel AC, Feuk L, Skaug J (2008). Structural variation of chromosomes in autism spectrum disorder. Am J Hum Genet.

[28] McInnes LA, Nakamine A, Pilorge M, Brandt T, Jimenez Gonzalez P, Fallas M (2010). A large-scale survey of the novel 15q24 microdeletion syndrome in autism spectrum disorders identifies an atypical deletion that narrows the critical region. Mol Autism.

[29] Siu WK, Lam CW, Gao WW, Tang VH, Jin DY, Mak CM (2015). Unmasking a novel disease gene NEO1 associated with autism spectrum disorders by a hemizygous deletion on chromosome 15 and a functional polymorphism. Behav Brain Res.

[30] Heard PL, Carter EM, Crandall AC, Sebold C, Hale DE, Cody JD (2009). High resolution genomic analysis of 18q- using oligo-microarray comparative genomic hybridization (aCGH). Am J Med Genet A.

[31] Cody JD, Hasi M, Soileau B, Heard P, Carter E, Sebold C (2014). Establishing a reference group for distal 18q-: clinical description and molecular basis. Hum Genet.

[32] Versacci P, Digilio MC, Sauer U, Dallapiccola B, Marino B (2005). Absent pulmonary valve with intact ventricular septum and patent ductus arteriosus: a specific cardiac phenotype associated with deletion 18q syndrome. Am J Med Genet A.

[33] van Trier DC, Feenstra I, Bot P, de Leeuw N, Draaisma JM (2013). Cardiac anomalies in individuals with the 18q deletion syndrome; report of a child with Ebstein anomaly and review of the literature. Eur J Med Genet.

[34] O’Donnell L, Soileau B, Heard P, Carter E, Sebold C, Gelfond J (2010). Genetic determinants of autism in individuals with deletions of 18q. Hum Genet.

[35] Ullmann R, Turner G, Kirchhoff M, Chen W, Tonge B, Rosenberg C (2007). Array CGH identifies reciprocal 16p13.1 duplications and deletions that predispose to autism and/or mental retardation. Hum Mutat.

[36] Ramalingam A, Zhou XG, Fiedler SD, Brawner SJ, Joyce JM, Liu HY (2011). 16p13.11 duplication is a risk factor for a wide spectrum of neuropsychiatric disorders. J Hum Genet.

[37] Ingason A, Rujescu D, Cichon S, Sigurdsson E, Sigmundsson T, Pietilainen OP (2011). Copy number variations of chromosome 16p13.1 region associated with schizophrenia. Mol Psychiatry.

[38] Williams NM, Zaharieva I, Martin A, Langley K, Mantripragada K, Fossdal R (2010). Rare chromosomal deletions and duplications in attention-deficit hyperactivity disorder: a genome-wide analysis. Lancet.

[39] Mefford HC, Cooper GM, Zerr T, Smith JD, Baker C, Shafer N (2009). A method for rapid, targeted CNV genotyping identifies rare variants associated with neurocognitive disease. Genome Res.

[40] Pinto D, Delaby E, Merico D, Barbosa M, Merikangas A, Klei L (2014). Convergence of genes and cellular pathways dysregulated in autism spectrum disorders. Am J Hum Genet.

[41] Hannes FD, Sharp AJ, Mefford HC, de Ravel T, Ruivenkamp CA, Breuning MH (2009). Recurrent reciprocal deletions and duplications of 16p13.11: the deletion is a risk factor for MR/MCA while the duplication may be a rare benign variant. J Med Genet.

[42] Tropeano M, Ahn JW, Dobson RJ, Breen G, Rucker J, Dixit A (2013). Male-biased autosomal effect of 16p13.11 copy number variation in neurodevelopmental disorders. PLoS ONE.

[43] Coe BP, Witherspoon K, Rosenfeld JA, van Bon BW, Vulto-van Silfhout AT, Bosco P (2014). Refining analyses of copy number variation identifies specific genes associated with developmental delay. Nat Genet.

[44] Cooper GM, Coe BP, Girirajan S, Rosenfeld JA, Vu TH, Baker C (2011). A copy number variation morbidity map of developmental delay. Nat Genet.

[45] Gaugler T, Klei L, Sanders SJ, Bodea CA, Goldberg AP, Lee AB (2014). Most genetic risk for autism resides with common variation. Nat Genet.

[46] Girirajan S, Dennis MY, Baker C, Malig M, Coe BP, Campbell CD (2013). Refinement and discovery of new hotspots of copy-number variation associated with autism spectrum disorder. Am J Hum Genet.

[47] Dauber A, Lafranchi SH, Maliga Z, Lui JC, Moon JE, McDeed C (2012). Novel microcephalic primordial dwarfism disorder associated with variants in the centrosomal protein ninein. J Clin Endocrinol Metab.

[48] Wang X, Tsai JW, Imai JH, Lian WN, Vallee RB, Shi SH (2009). Asymmetric centrosome inheritance maintains neural progenitors in the neocortex. Nature.

[49] Srivatsa S, Parthasarathy S, Molnar Z, Tarabykin V (2015). Sip1 downstream Effector ninein controls neocortical axonal growth, ipsilateral branching, and microtubule growth and stability. Neuron.

[50] Blockus H, Chedotal A (2014). The multifaceted roles of Slits and Robos in cortical circuits: from proliferation to axon guidance and neurological diseases. Curr Opin Neurobiol.

[51] Chu J, Anderson SA (2015). Development of cortical interneurons. Neuropsychopharmacology.

[52] Marin O (2012). Interneuron dysfunction in psychiatric disorders. Nat Rev Neurosci.

[53] Robichaux MA, Cowan CW (2014). Signaling mechanisms of axon guidance and early synaptogenesis. Curr Top Behavl Neurosci.

[54] Alarcon M, Abrahams BS, Stone JL, Duvall JA, Perederiy JV, Bomar JM (2008). Linkage, association, and gene-expression analyses identify CNTNAP2 as an autism-susceptibility gene. Am J Hum Genet.

[55] Penagarikano O, Abrahams BS, Herman EI, Winden KD, Gdalyahu A, Dong H (2011). Absence of CNTNAP2 leads to epilepsy, neuronal migration abnormalities, and core autism-related deficits. Cell.

[56] Zweier C, de Jong EK, Zweier M, Orrico A, Ousager LB, Collins AL (2009). CNTNAP2 and NRXN1 are mutated in autosomal-recessive Pitt-Hopkins-like mental retardation and determine the level of a common synaptic protein in Drosophila. Am J Hum Genet.

[57] Ramocki MB, Tavyev YJ, Peters SU (2010). The MECP2 duplication syndrome. Am J Med Genet A.

