# Supplementary material for: Diagnostic yield of array CGH in patients with autism spectrum disorder in Hong Kong
Source: Clin Transl Med. 2016 May 16;5:18. doi: 10.1186/s40169-016-0098-1 (PMC4896892; doi:10.1186/s40169-016-0098-1)

Supplementary Information for "Diagnostic yield of Array CGH in Patients with Autism Spectrum Disorder in Hong Kong"

Wai-Kwan Siu[1,2], Ching-Wan Lam[1*], Chloe Miu Mak[2], Elizabeth Tak-Kwong Lau[3], Mary Hoi-Yin Tang[3], Wing-Fai Tang[3], Rachel Sui-Man Poon-Mak[4], Chi-Chiu Lee[5], Se-Fong Hung[5], Patrick Wing-Leung Leung[6], Karen Ling Kwong[7], Eric Kin-Cheong Yau[8], Grace Sui-Fun Ng[8], Nai-Chung Fong[8], Kwok-Yin Chan[8]

[1] Department of Pathology, The University of Hong Kong, Queen Mary Hospital, Hong Kong, China

[2] Kowloon West Cluster Laboratory Genetics Service, Department of Pathology, Princess Margaret Hospital, Hong Kong, China

[3] Department of Obstetrics & Gynaecology, The University of Hong Kong, Queen Mary Hospital, Hong Kong, China

[4] Department of Clinical Psychology, Kwai Chung Hospital, Hong Kong, China

[5] Department of Psychiatry, Kwai Chung Hospital, Hong Kong, China

[6] Department of Psychology, The Chinese University of Hong Kong, Hong Kong, China

[7] Department of Paediatrics & Adolescent Medicine, Tuen Mun Hospital, Hong Kong, China

[8] Department of Paediatrics & Adolescent Medicine, Princess Margaret Hospital, Hong Kong, China

List of Figures

Figure S1 Array CGH showing 1.16 Mb duplication at 16p13.11 in Patients 1, 4, 5 & 8 3

Figure S2 Array CGH showing 0.26 Mb deletion at 15q11.2 in Patient 2 4

Figure S3 Array CGH showing 1.97 Mb deletion at 15q23–q24.1in Patient 3 5

Figure S4 Array CGH showing 0.12 Mb deletion at 14q22.1 in Patient 6 6

Figure S5 Array CGH showing 14.53 Mb deletion at 18q22.1–q23 in Patient 7 7

Figure S6 Array CGH showing 0.55 Mb duplication at 3q13.3 in the subject Patient 9 8

Figure S7 Array CGH showing 0.61 Mb duplication at 1q44 in Patient 10 9

Figure S8 Array CGH showing 0.08 Mb duplication at 11q24.1 in Patient 11 10

Figure S9 Array CGH showing 0.97 Mb duplication at 10p12.33–p12.32 in Patient 12 11

Figure S10 Array CGH showing 0.12 Mb duplication at 17q21.33 in Patient 13 ..12

Figure S11 Array CGH showing 0.64 Mb duplication at 6q14.1 in Patient 14 13

Figure S12 Array CGH showing 0.58 Mb deletion at 5q33.1 in Patient 15 14

Figure S13 Chromosome 18q deletion...15

Figure S1 Array CGH showing 1.16 Mb duplication at 16p13.11 in Patients 1, 4, 5 & 8

The scatterplot of chromosome view is displayed using DEVA software. The first track is the cytogenetic ideaogram of chromosome 16 and the second to the fifth tracks are the log_2 ratio data of Patients 1, 4, 5 and 8 respectively. The X axis represents the coordinate on chromosome 16 and the Y axis repsents the log_2 ratio. The red dots indcate the probes with log_2 ratio > 0 and blue dots denote probes with log_2 ratio ≤ 0. The area enclosed by the dashbox are enlarged below.

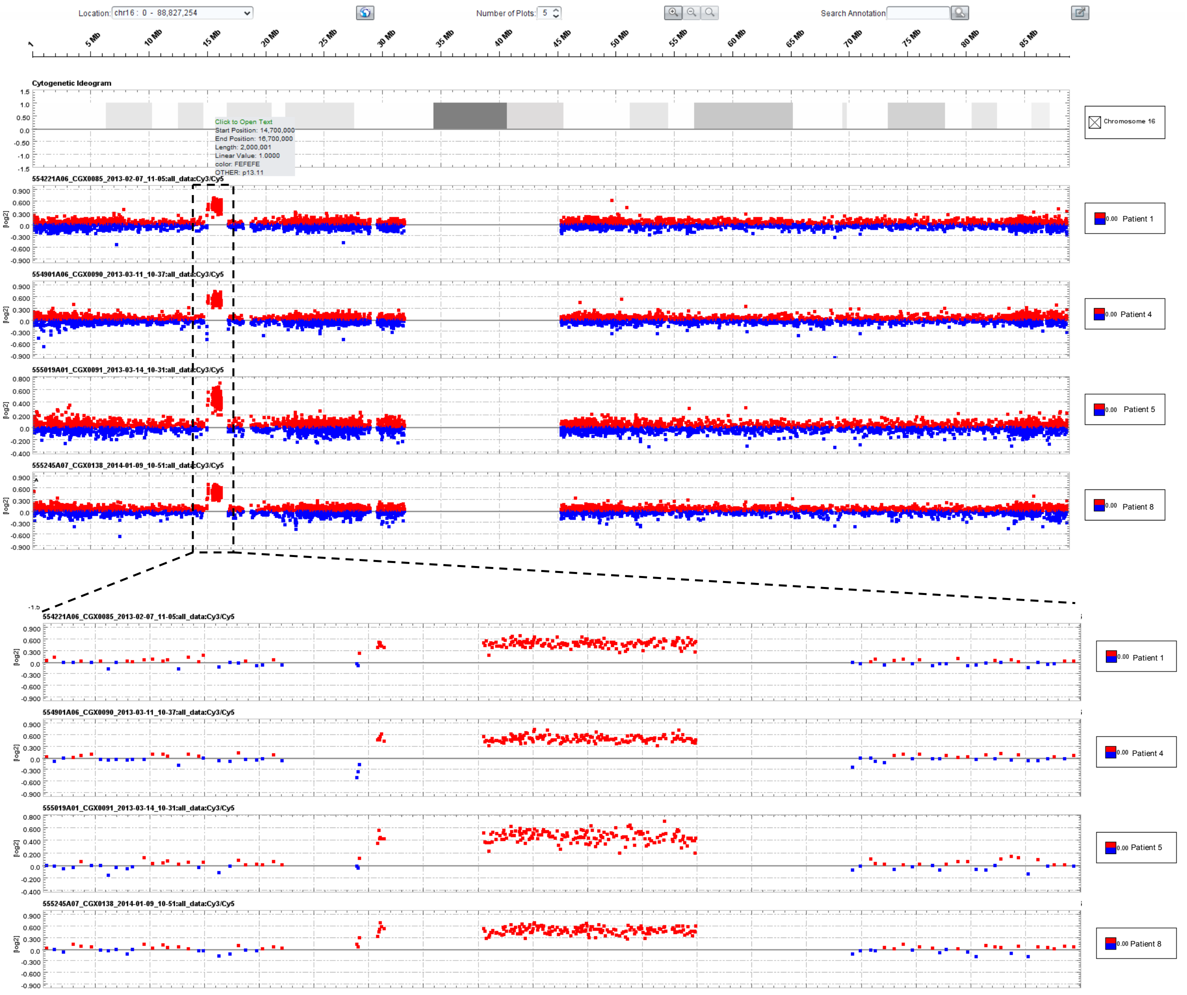

Figure S2 Array CGH showing 0.26 Mb deletion at 15q11.2 in Patient 2

The scatterplot of chromosome view is displayed using DEVA software. The first track is the cytogenetic ideaogram of chromosome 15 and the second track is the $\log_2$ ratio data of Patient 2. The X axis represents the coordinate on chromosome 15 and the Y axis repsents the $\log_2$ ratio. The red dots indcate the probes with $\log_2$ ratio >0 and blue dots denote probes with $\log_2$ ratio ≤ 0. The area enclosed by the dashbox are enlarged below.

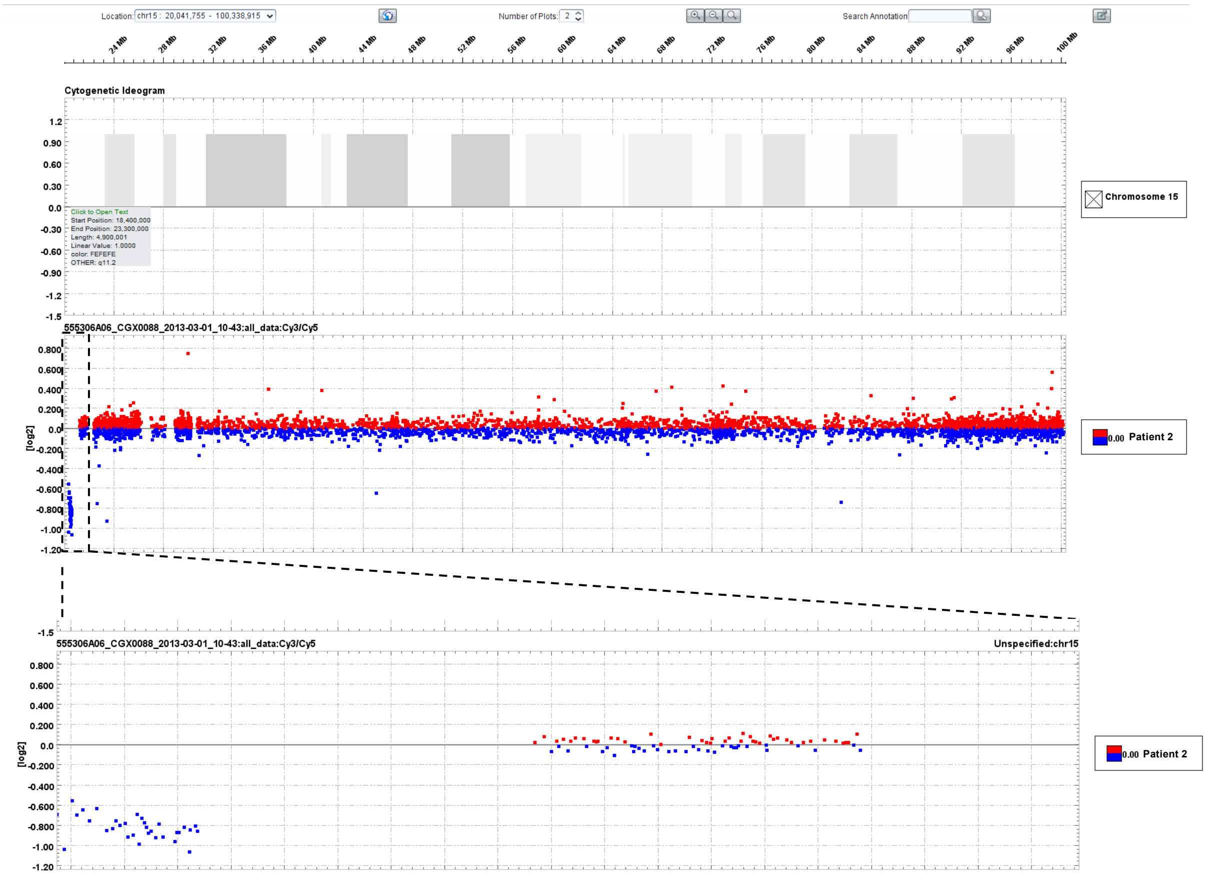

Figure S3 Array CGH showing 1.97 Mb deletion at 15q23–q24.1in Patient 3

The scatterplot of chromosome view is displayed using DEVA software. The first track is the cytogenetic ideaogram of chromosome 15 and the second track is the $\log_2$ ratio data of Patient 3. The X axis represents the coordinate on chromosome 15 and the Y axis repsents the $\log_2$ ratio. The red dots indcate the probes with $\log_2$ ratio > 0 and blue dots denote probes with $\log_2$ ratio ≤ 0. The area enclosed by the dashbox are enlarged below.

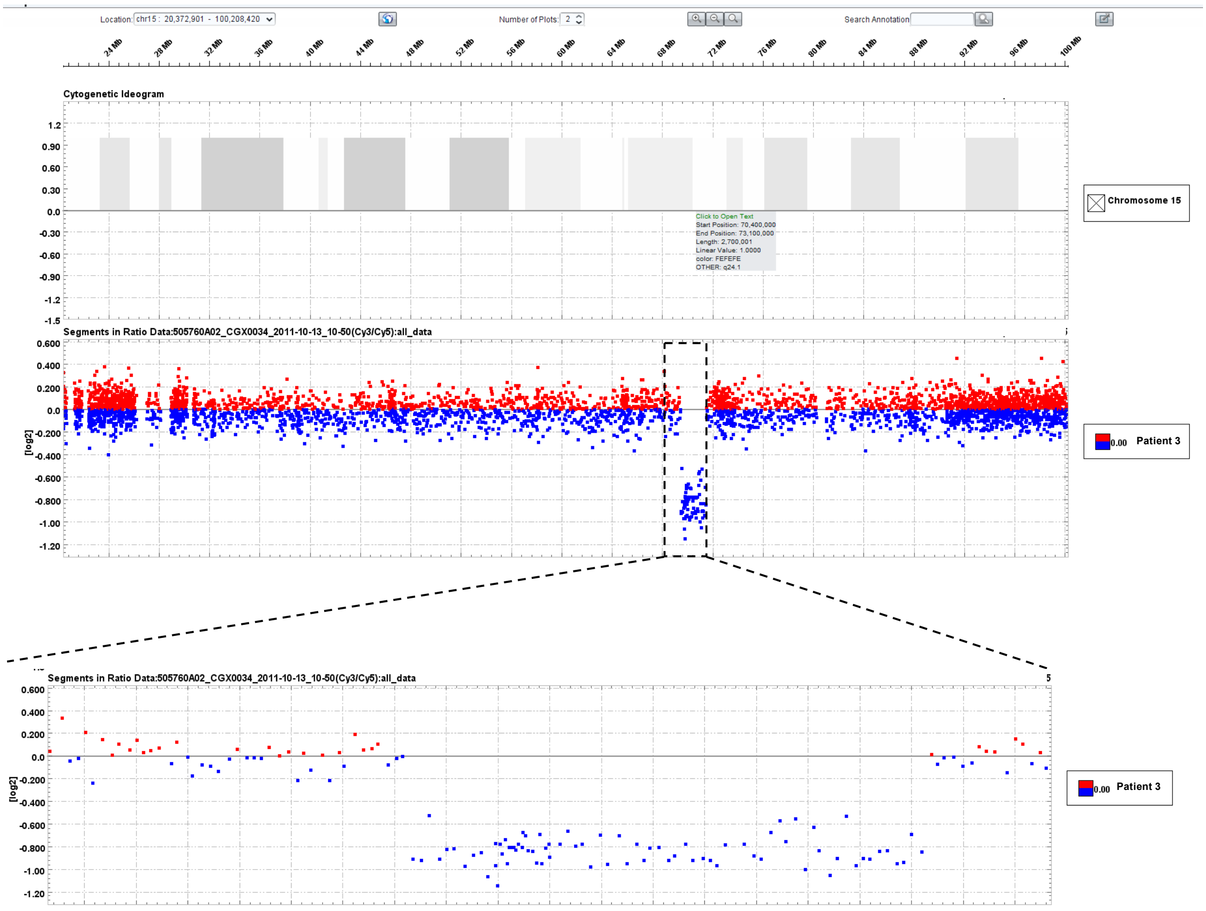

Figure S4 Array CGH showing 0.12 Mb deletion at 14q22.1 in Patient 6

The scatterplot of chromosome view is displayed using DEVA software. The first track is the cytogenetic ideaogram of chromosome 14 and the second track is the $\log_2$ ratio data of Patient 6. The X axis represents the coordinate on chromosome 14 and the Y axis repsents the $\log_2$ ratio. The red dots indcate the probes with $\log_2$ ratio >0 and blue dots denote probes with $\log_2$ ratio ≤ 0. The area enclosed by the dashbox are enlarged below with an additional track showing the gene in the region of deleton.

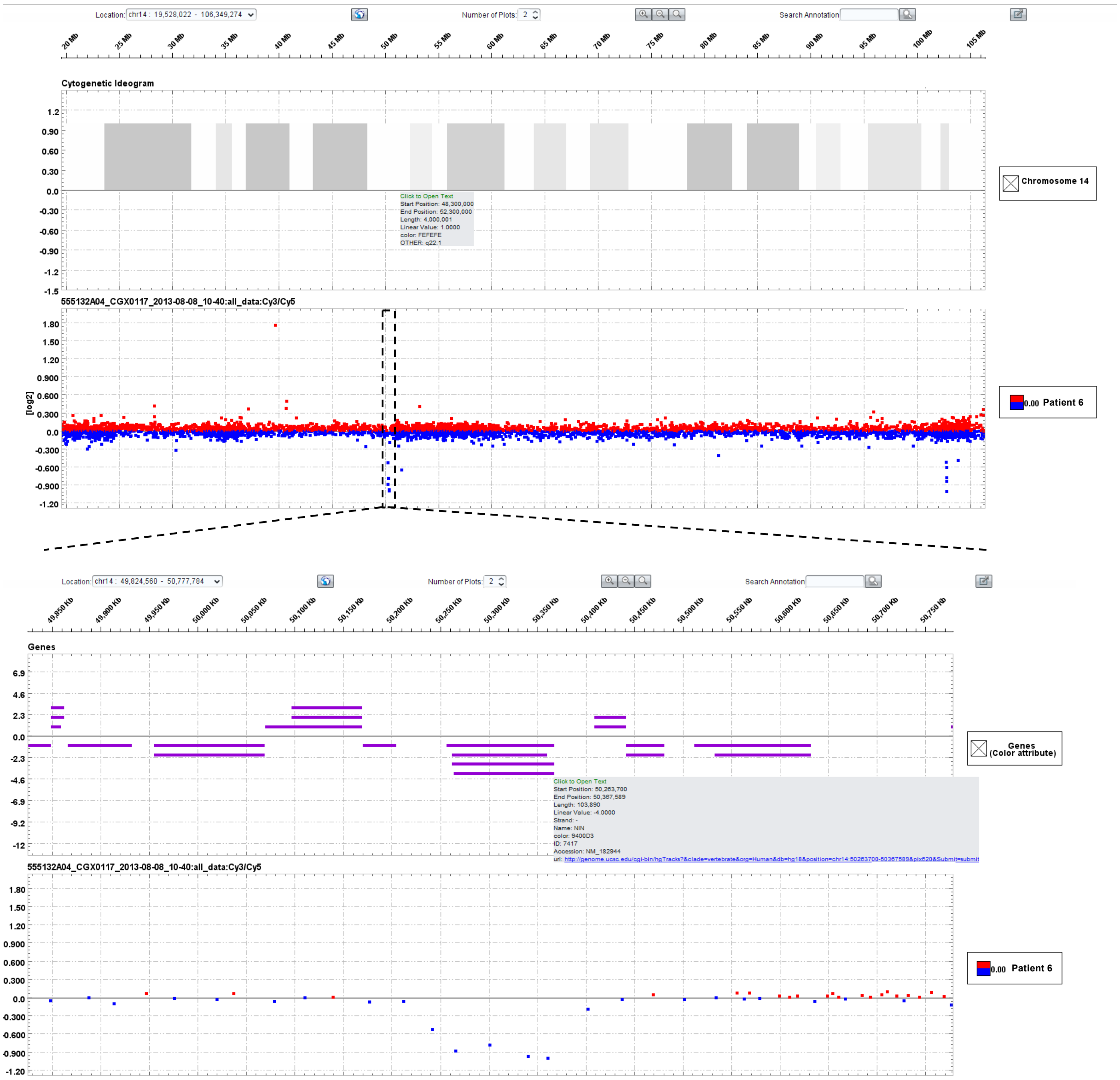

Figure S5 Array CGH showing 14.53 Mb deletion at 18q22.1–q23 in Patient 7

The scatterplot of chromosome view is displayed using DEVA software. The first track is the cytogenetic ideaogram of chromosome 18 and the second track is the $\log_2$ ratio data of Patient 7. The X axis represents the coordinate on chromosome 18 and the Y axis repsents the $\log_2$ ratio. The red dots indcate the probes with $\log_2$ ratio > 0 and blue dots denote probes with $\log_2$ ratio ≤ 0. The area enclosed by the dashbox are enlarged below.

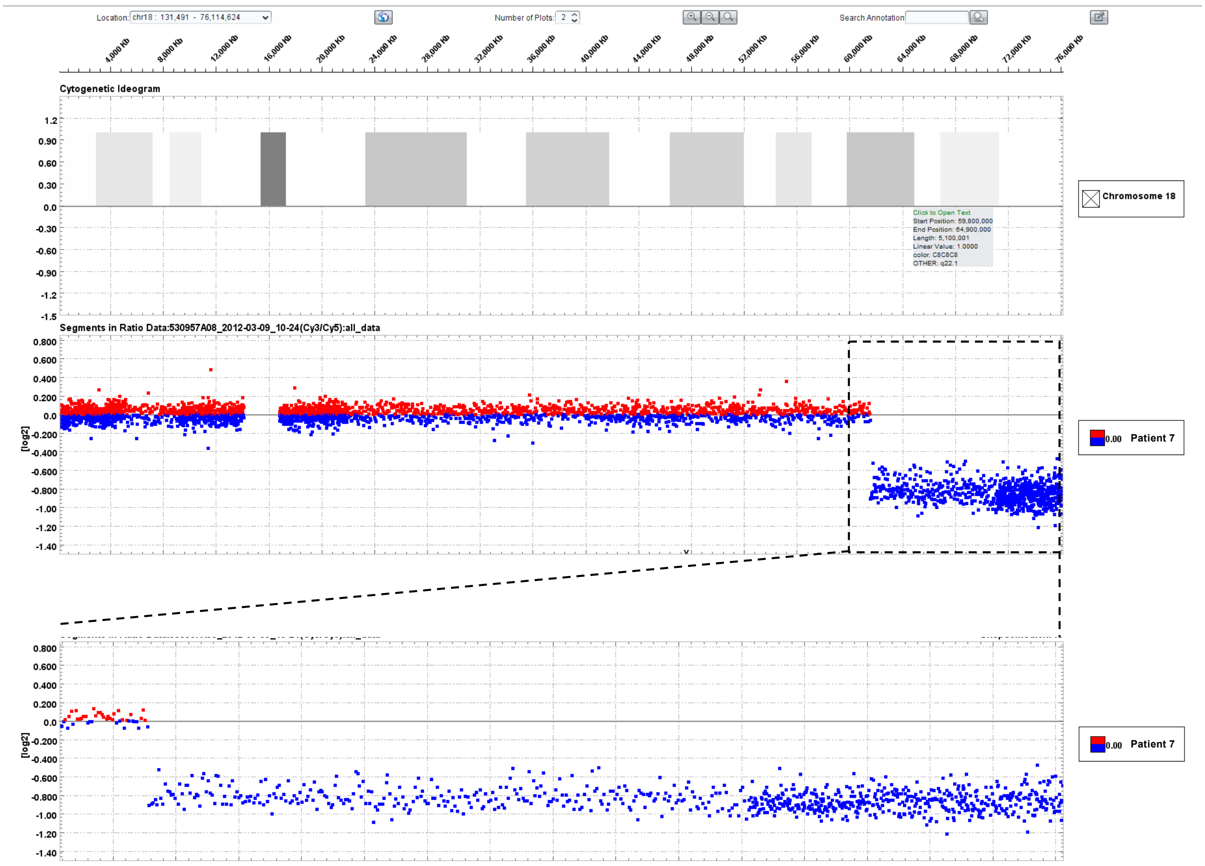

Figure S6 Array CGH showing 0.55 Mb duplication at 3q13.3 in the subject Patient 9

The scatterplot of chromosome view is displayed using DEVA software. The first track is the cytogenetic ideaogram of chromosome 3 and the second track is the log_2 ratio data of Patient 9. The X axis represents the coordinate on chromosome 3 and the Y axis repsents the log_2 ratio. The red dots indcate the probes with log_2 ratio > 0 and blue dots denote probes with log_2 ratio ≤ 0. The area enclosed by the dashbox are enlarged below.

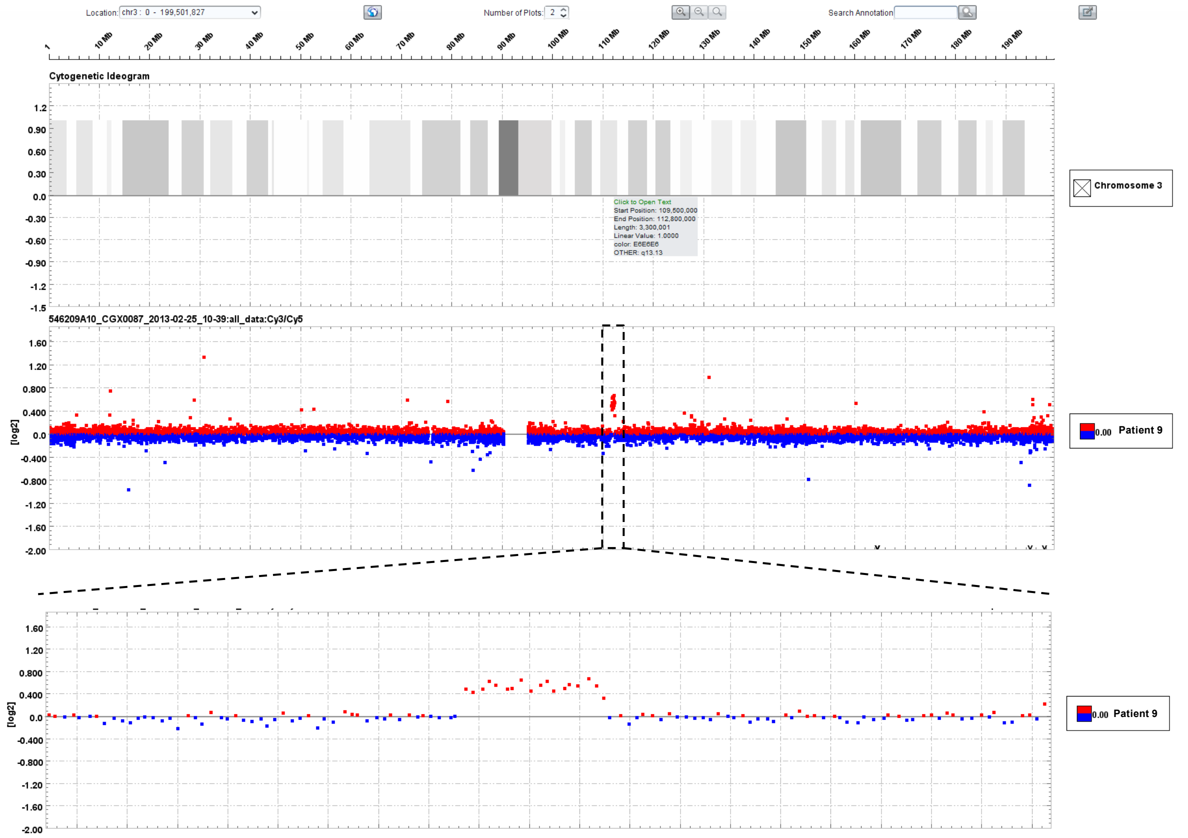

Figure S7 Array CGH showing 0.61 Mb duplication at 1q44 in Patient 10

The scatterplot of chromosome view is displayed using DEVA software. The first track is the cytogenetic ideaogram of chromosome 1 and the second track is the $\log_2$ ratio data of Patient 10. The X axis represents the coordinate on chromosome 1 and the Y axis repsents the $\log_2$ ratio. The red dots indcate the probes with $\log_2$ ratio > 0 and blue dots denote probes with $\log_2$ ratio ≤ 0. The area enclosed by the dashbox are enlarged below.

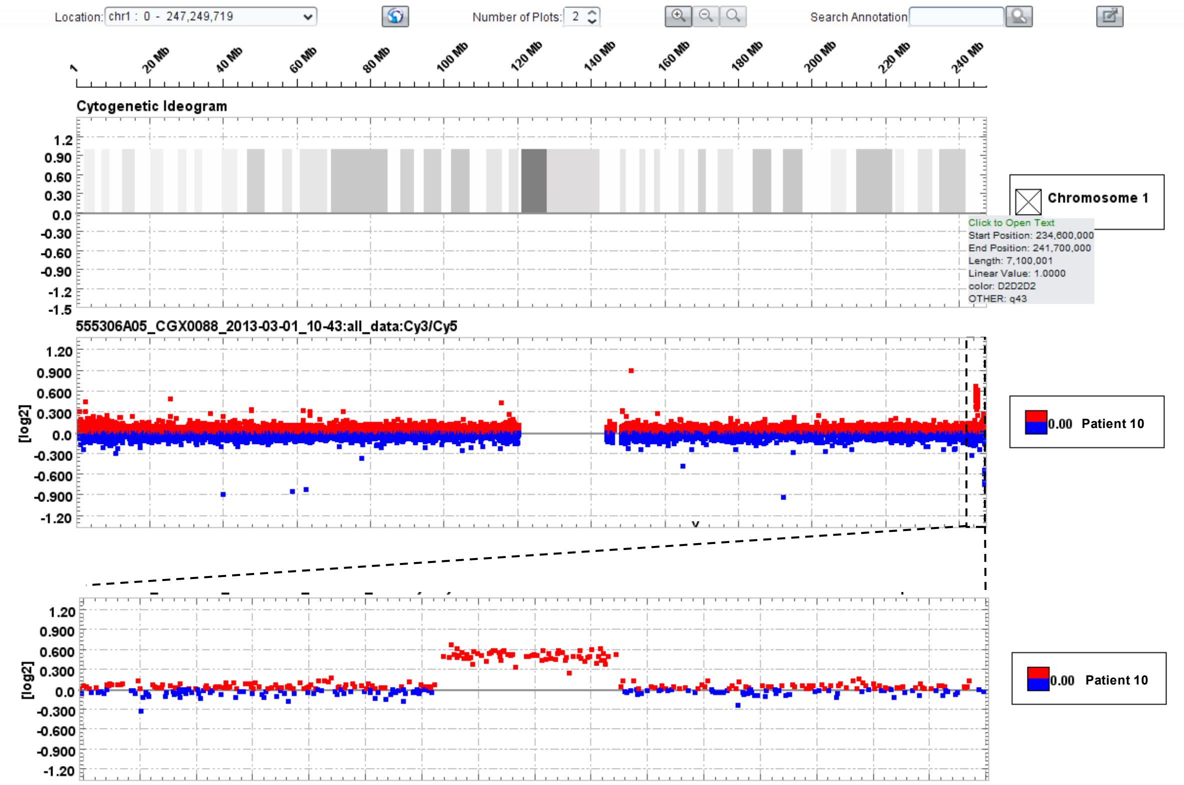

Figure S8 Array CGH showing 0.08 Mb duplication at 11q24.1 in Patient 11

The scatterplot of chromosome view is displayed using DEVA software. The first track is the cytogenetic ideaogram of chromosome 11 and the second track is the $\log_2$ ratio data of Patient 11. The X axis represents the coordinate on chromosome 11 and the Y axis repsents the $\log_2$ ratio. The red dots indcate the probes with $\log_2$ ratio > 0 and blue dots denote probes with $\log_2$ ratio ≤ 0. The area enclosed by the dashbox are enlarged below.

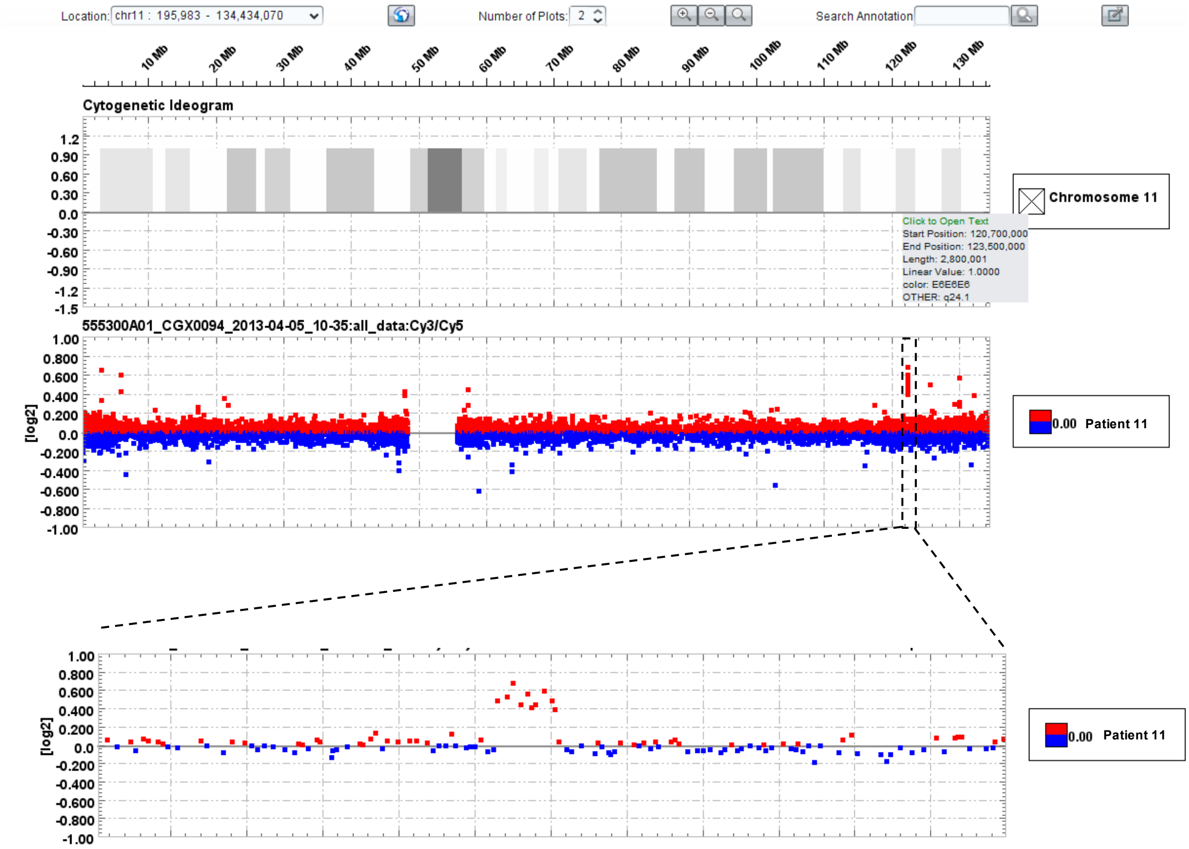

Figure S9 Array CGH showing 0.97 Mb duplication at 10p12.33–p12.32 in Patient 12

The scatterplot of chromosome view is displayed using DEVA software. The first track is the cytogenetic ideaogram of chromosome 10 and the second track is the $\log_2$ ratio data of Patient 12. The X axis represents the coordinate on chromosome 10 and the Y axis repsents the $\log_2$ ratio. The red dots indcate the probes with $\log_2$ ratio > 0 and blue dots denote probes with $\log_2$ ratio ≤ 0. The area enclosed by the dashbox are enlarged below.

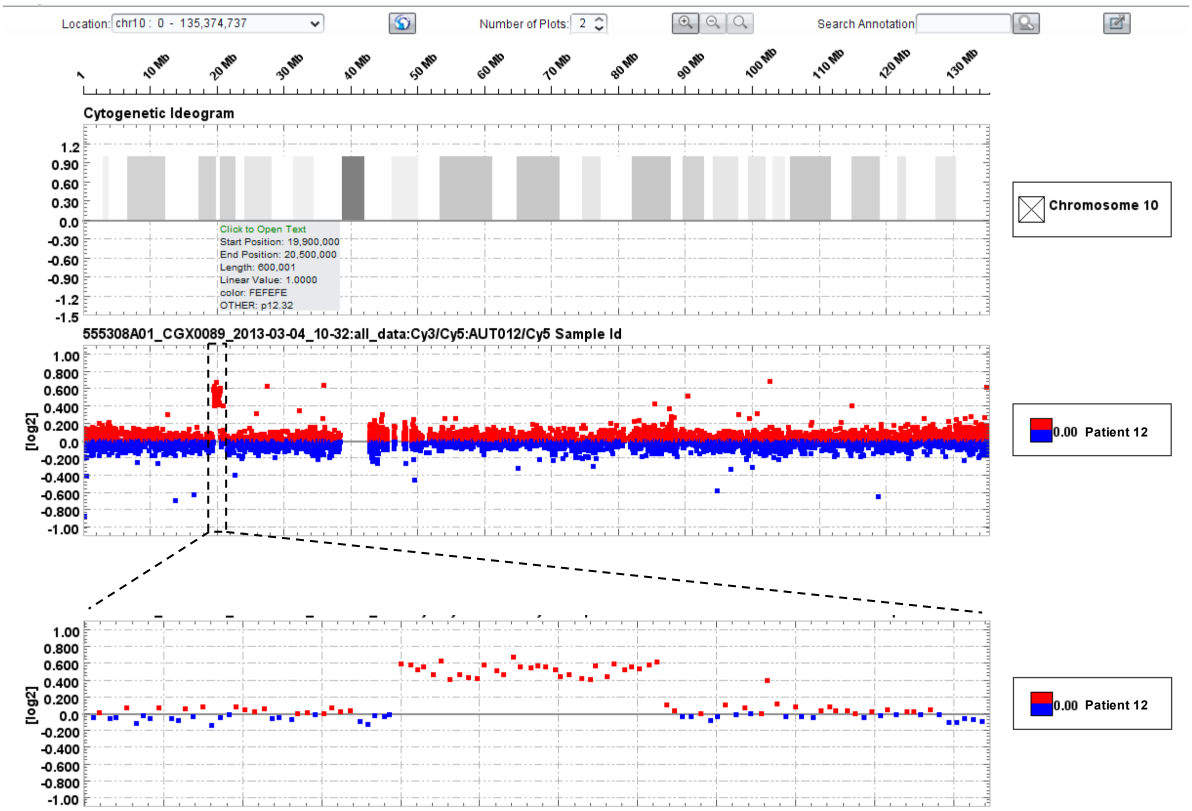

Figure S10 Array CGH showing 0.12 Mb duplication at 17q21.33 in Patient 13

The scatterplot of chromosome view is displayed using DEVA software. The first track is the cytogenetic ideaogram of chromosome 17 and the second track is the log_2 ratio data of Patient 13. The X axis represents the coordinate on chromosome 17 and the Y axis repsents the log_2 ratio. The red dots indcate the probes with log_2 ratio > 0 and blue dots denote probes with log_2 ratio ≤ 0. The area enclosed by the dashbox are enlarged below.

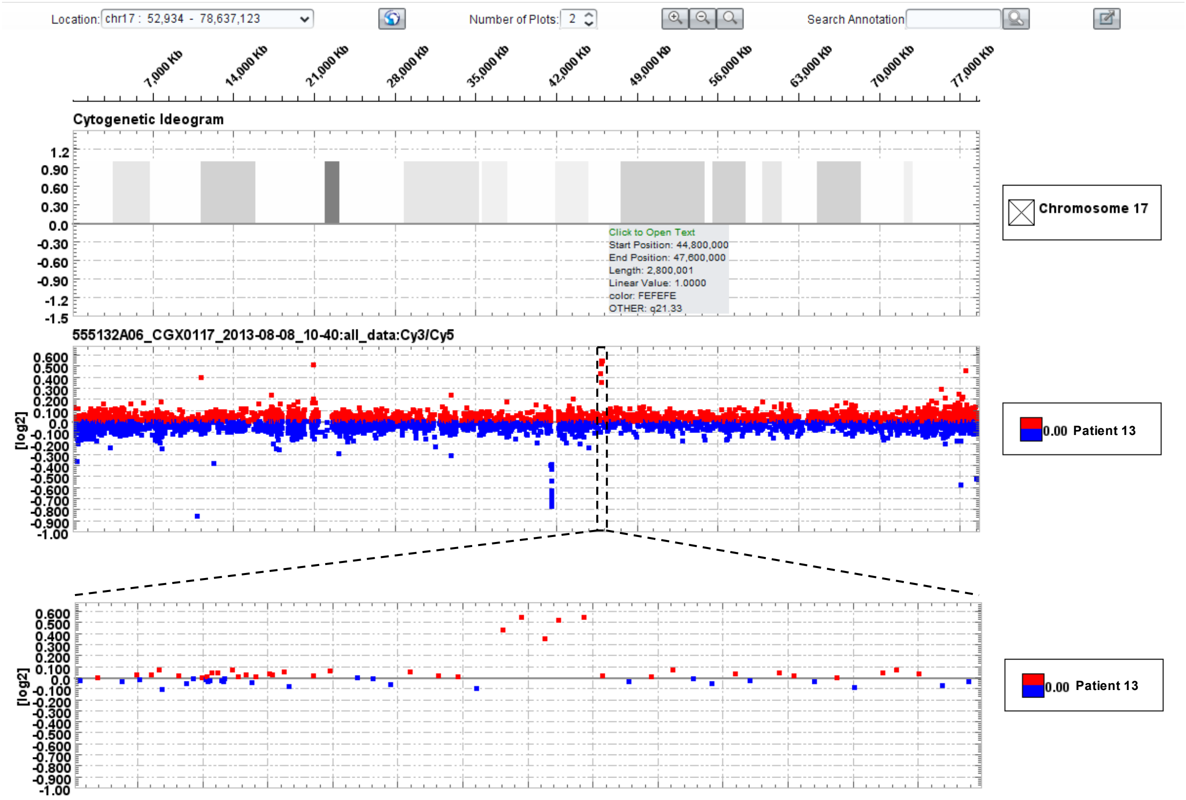

Figure S11 Array CGH showing 0.64 Mb duplication at 6q14.1 in Patient 14

The scatterplot of chromosome view is displayed using DEVA software. The first track is the cytogenetic ideaogram of chromosome 6 and the second track is the log_2 ratio data of Patient 14. The X axis represents the coordinate on chromosome 6 and the Y axis repsents the log_2 ratio. The red dots indcate the probes with log_2 ratio > 0 and blue dots denote probes with log_2 ratio ≤ 0. The area enclosed by the dashbox are enlarged below.

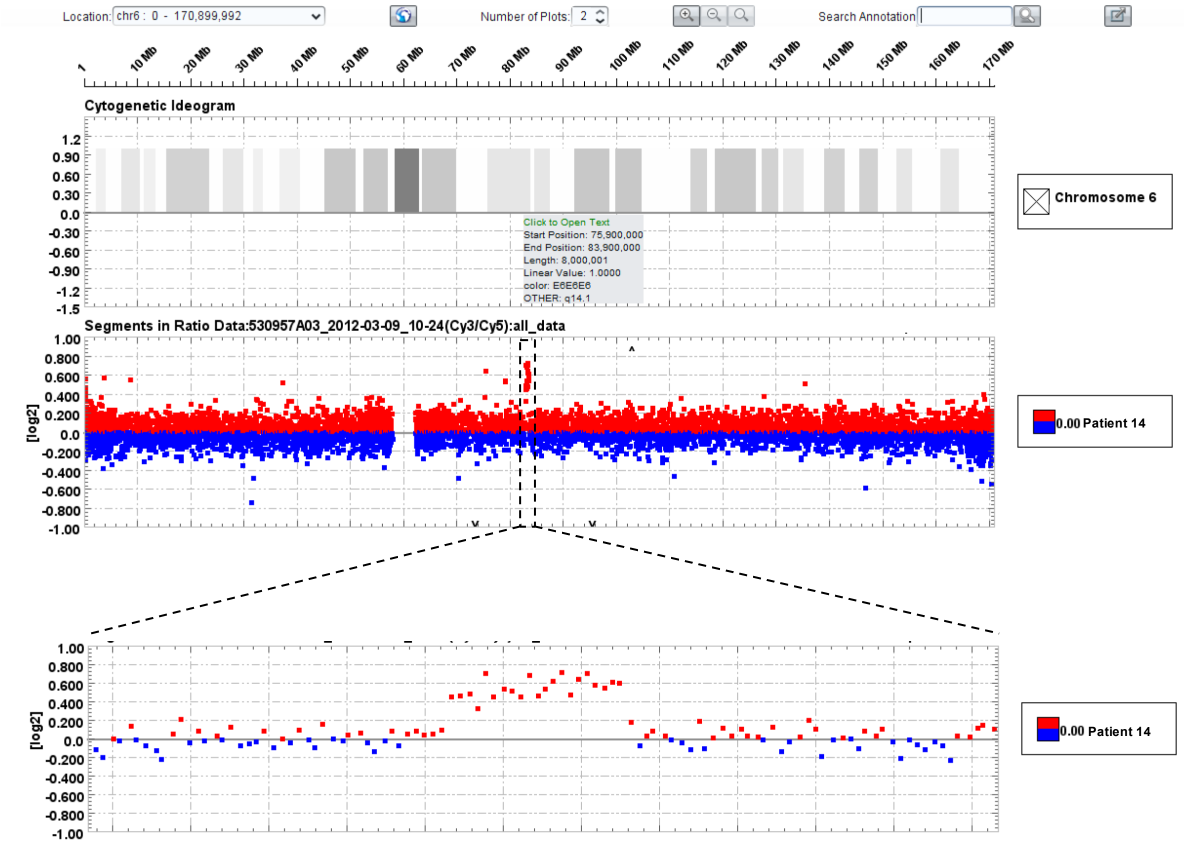

Figure S12 Array CGH showing 0.58 Mb deletion at 5q33.1 in Patient 15

The scatterplot of chromosome view is displayed using DEVA software. The first track is the cytogenetic ideaogram of chromosome 5 and the second track is the $\log_2$ ratio data of Patient 15. The X axis represents the coordinate on chromosome 5 and the Y axis repsents the $\log_2$ ratio. The red dots indcate the probes with $\log_2$ ratio > 0 and blue dots denote probes with $\log_2$ ratio ≤ 0. The area enclosed by the dashbox are enlarged below.

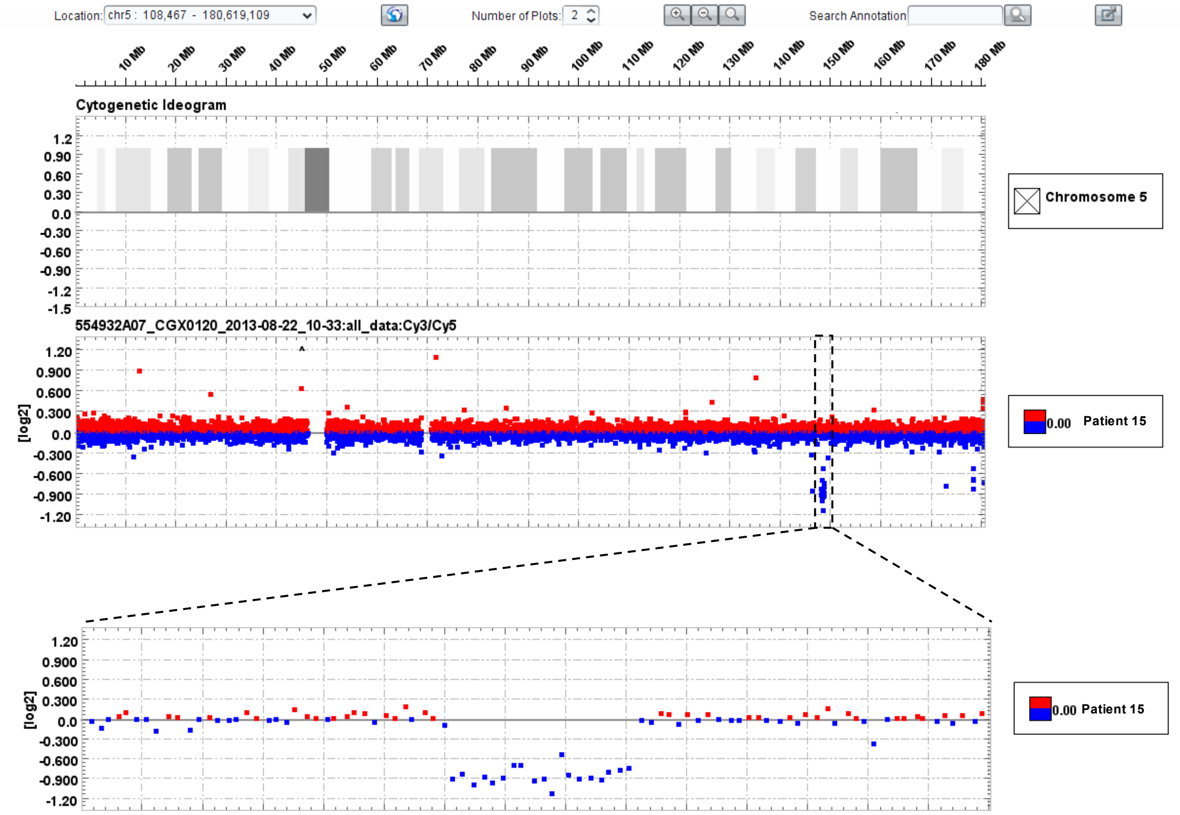

Figure S13 Chromosome 18q deletion

The 18q deletion in Patient 7 is shown as UCSC custom track. The chromosome 18 ideogram is displayed at the top of the figure with a red box specifying the region delineated below. The blue bar indicates the hemizgyous 18q deletion present in Patient 7. The locations of RefSeq genes are shown in the bottom with *NETO1* and *FBXO15*, genes in 18q region associated with increased risk of autism, highlighted by red circle.

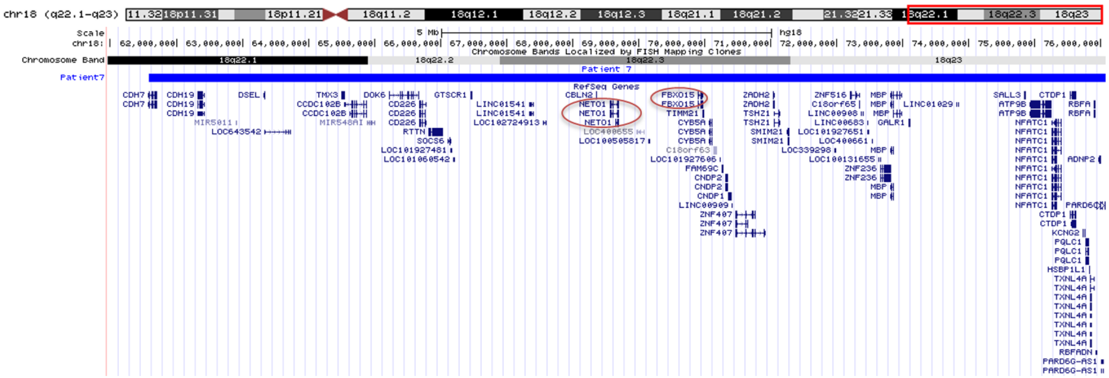

Supplement: Supplementary file 1 — 10.1186/s40169-016-0098-1 Figures of array CGH data. [file 40169_2016_98_MOESM1_ESM.pdf]
